# Collapse of Insect Gut Symbiosis under Simulated Climate Change

**DOI:** 10.1128/mBio.01578-16

**Published:** 2016-10-04

**Authors:** Yoshitomo Kikuchi, Akiyo Tada, Dmitry L. Musolin, Nobuhiro Hari, Takahiro Hosokawa, Kenji Fujisaki, Takema Fukatsu

**Affiliations:** a National Institute of Advanced Industrial Science and Technology (AIST) Hokkaido, Sapporo, Japan; b Graduate School of Agriculture, Kyoto University, Kyoto, Japan; c Saint Petersburg State Forest Technical University, Saint Petersburg, Russia; d Faculty of Science, Kyushu University, Fukuoka, Japan; e National Institute of Advanced Industrial Science and Technology (AIST), Tsukuba, Japan

## Abstract

Global warming impacts diverse organisms not only directly but also indirectly via other organisms with which they interact. Recently, the possibility that elevated temperatures resulting from global warming may substantially affect biodiversity through disrupting mutualistic/parasitic associations has been highlighted. Here we report an experimental demonstration that global warming can affect a pest insect via suppression of its obligate bacterial symbiont. The southern green stinkbug *Nezara viridula* depends on a specific gut bacterium for its normal growth and survival. When the insects were reared inside or outside a simulated warming incubator wherein temperature was controlled at 2.5°C higher than outside, the insects reared in the incubator exhibited severe fitness defects (i.e., retarded growth, reduced size, yellowish body color, etc.) and significant reduction of symbiont population, particularly in the midsummer season, whereas the insects reared outside did not. Rearing at 30°C or 32.5°C resulted in similar defective phenotypes of the insects, whereas no adult insects emerged at 35°C. Notably, experimental symbiont suppression by an antibiotic treatment also induced similar defective phenotypes of the insects, indicating that the host’s defective phenotypes are attributable not to the heat stress itself but to the suppression of the symbiont population induced by elevated temperature. These results strongly suggest that high temperature in the midsummer season negatively affects the insects not directly but indirectly via the heat-vulnerable obligate bacterial symbiont, which highlights the practical relevance of mutualism collapse in this warming world.

## IMPORTANCE

Climate change is among the biggest environmental issues in the contemporary world, and its impact on the biodiversity and ecosystem is not only of scientific interest but also of practical concern for the general public. On the basis of our laboratory data obtained under strictly controlled environmental conditions and our simulated warming data obtained in seminatural settings (elevated 2.5°C above the normal temperature), we demonstrate here that *Nezara viridula*, the notorious stinkbug pest, suffers serious fitness defects in the summer season under the simulated warming conditions, wherein high temperature acts on the insect not directly but indirectly via suppression of its obligate gut bacterium. Our finding highlights that heat-susceptible symbionts can be the “Achilles’ heel” of symbiont-dependent organisms under climate change conditions.

## INTRODUCTION

Elevated temperature associated with global climate change impacts diverse organisms not only directly but also indirectly through other organisms with which they interact (1–4). Recent studies have emphasized the possibility that global climate change can substantially affect the biodiversity and ecosystem via disruption of mutualistic/parasitic associations such as coral-dinoflagellate mutualism, plant-pollinator mutualism, amphibian-trematode parasitism, and many others (5–8). A number of animals and plants harbor symbiotic microorganisms inside their bodies, wherein the symbionts play pivotal roles in the metabolism of their hosts (9, 10), but empirical investigations of how global climate change affects these symbiotic associations have been scarce.

**FIG 1:**
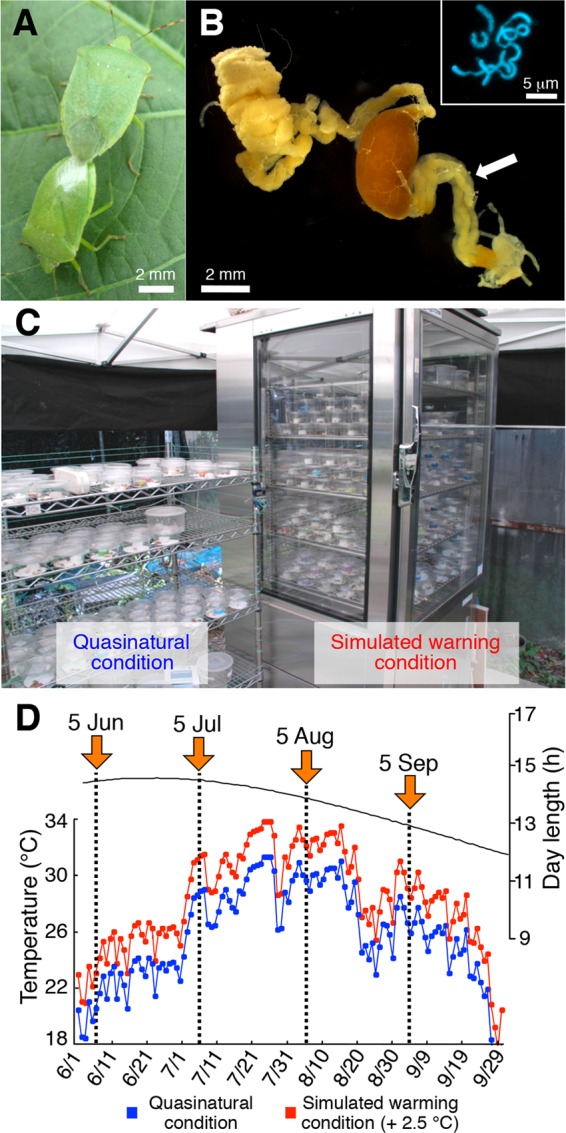
The southern green stinkbug *N. viridula* and simulated warming experimental system. (A) A mating pair of *N. viridula* stinkbugs on a soybean leaf. (B) A dissected midgut of the insect. An arrow indicates the symbiotic organ bearing a number of crypts, which are filled with symbiotic bacteria (see inset). (C) The incubator for the simulated warming condition and adjacent open metal shelves for the quasinatural condition. These facilities were set outdoors, with only a canopy tent cover for protection from direct sunlight and rain. (D) Environmental conditions during the experiment: black line, natural day length; blue line, temperature under the quasinatural condition; red line, temperature under the simulated warming condition. Arrows indicate the dates when egg masses of the insect were allocated to the experimental conditions (Jun, June; Jul, July, Aug, August; Sep, September). Note that the SW condition mimicked the QN condition except that the temperature was 2.5°C higher.

The southern green stinkbug *Nezara viridula* (Fig. 1A), a notorious insect pest infesting diverse crop plants worldwide (11), is associated with a specific gammaproteobacterial symbiont in a posterior region of the midgut (Fig. 1B). Upon oviposition, females of *N. viridula* smear symbiont-containing excretion onto the egg surface, which newborn nymphs orally acquire to establish vertical transmission of the symbiont (12). Elimination of the symbiont by egg surface sterilization causes severe fitness defects, such as retarded nymphal growth and elevated nymphal mortality, with few juveniles reaching adulthood (12). These results indicate that the obligate nature of the symbiotic association is likely based on provisioning of nutritional components deficient in plant sap diet (12, 13).

Field monitoring of *N. viridula* in mainland Japan has shown that its northern distribution limit had shifted northward by >85 km during the past 50 years (14–16), which has been discussed as a potential case of expanded distribution range northward in association with global warming (17, 18). *N. viridula* is easily maintained in the laboratory and is experimentally tractable (11, 18, 19), providing an ideal opportunity to investigate the field observations in the context of experimental settings simulating current and future climate change (6, 20). In this study, using a special rearing incubator whose inner temperature is controllable with reference to outside temperature, we experimentally investigated biological effects of future global warming on *N. viridula* and its gut symbiotic bacterium.

## RESULTS AND DISCUSSION

### Fitness defects under simulated warming conditions in midsummer.

**FIG 2:**
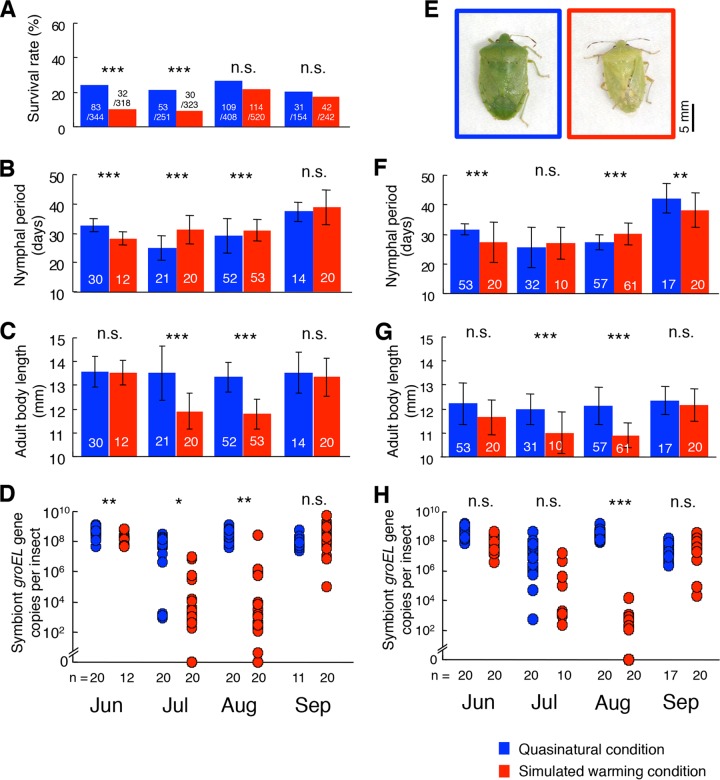
Effects of the simulated warming condition on *N. viridula* and its symbiont population. (A) Survival rate (i.e., proportion of the eggs that reached adulthood, including both sexes). (B to E) Female insects: (B) nymphal period; (C) adult body length; (D) symbiont titer; (E) typical body color of adult insects that emerged in August. (F to H) Male insects: (F) nymphal period; (G) adult body length; (H) symbiont titer. Blue and red indicate the QN condition and the SW condition, respectively. Means and standard deviations are shown in panels B, C, F, and G. Asterisks indicate statistically significant differences (*, *P* < 0.01; **, *P* < 0.001; ***, *P* < 0.0001). n.s., not statistically significant. Sample sizes are shown on the bars or below the plots.

We collected egg masses of an *N. viridula* strain derived from the current northern limit of the species and reared the sibling insects inside and outside the simulated warming incubator that was placed outdoors (Fig. 1C and D). Within the incubator, temperature was controlled at 2.5°C higher than outside, which is close to the average of the temperature increase range of global warming predicted to occur by 2100 (i.e., 0.3°C to 4.8°C) (21). Fresh egg masses were divided and randomly allocated to either the quasinatural (QN) condition (i.e., outside the incubator) or the simulated warming (SW) condition (i.e., inside the incubator) on 5 June, 5 July, 5 August, and 5 September 2009 (Fig. 1D; see also Fig. S1A in the supplemental material). Nymphs hatched soon, and their fitness parameters, including survival rate (i.e., survival rate from hatching to adult emergence), nymphal period (i.e., period from hatching to adult emergence), adult body length, and adult body coloration, were recorded. Overall, the fitness parameters tended to be significantly different between the QN condition and the SW condition, whereas the patterns contrasted between the moderately warm early summer/autumn (June/September) and hot midsummer (July/August) (Fig. 2). Specifically, under the SW condition, the nymphal period tended to be shorter in June and September, whereas it tended to be longer in July and August (Fig. 2B and F), and adult body size tended to be smaller in July and August, whereas no size differences were detected between the treatments in June and September (Fig. 2C and G). In addition to the reduced body size, many adult insects reared under the SW condition in midsummer exhibited yellowish abnormal body coloration, whereas those reared under the QN condition in midsummer were normally green in color (Fig. 2E; see also Fig. S2). These patterns, namely, a prolonged nymphal period, a reduced body size, and abnormal body coloration under the SW condition in midsummer, were concordant with those reported in our previous study (22) in that the effects of the simulated warming were deleterious in the midsummer season.

### Fitness defects under high temperature conditions in the laboratory.

**FIG 3:**
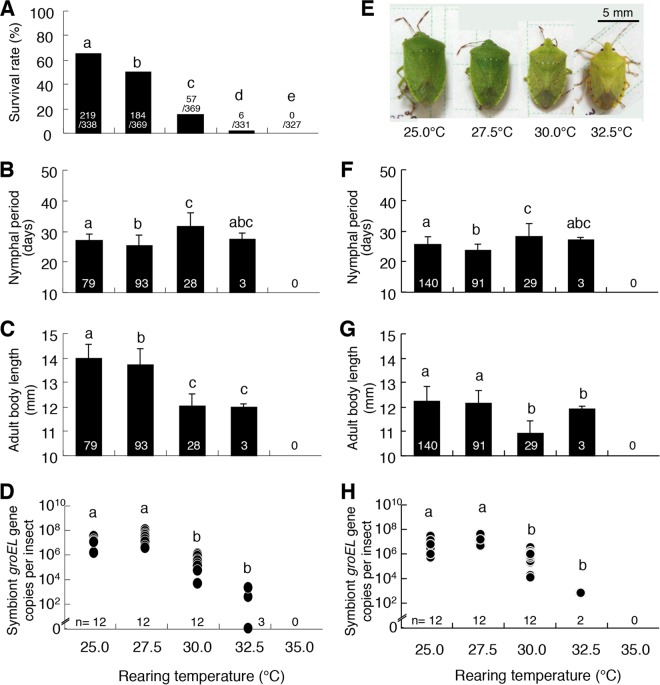
Effects of the constant temperature conditions on *N. viridula* and its symbiont population. (A) Survival rate (i.e., proportion of the eggs that reached adulthood, including both sexes). (B to E) Female insects: (B) nymphal period; (C) adult body length; (D) symbiont titer; (E) typical body color. (F to H) Male insects: (F) nymphal period; (G) adult body length; (H) symbiont titer. Means and standard deviations are shown in panels B, C, F, and G. Different letters indicate statistically significant differences (*P* < 0.05). Sample sizes are shown on the bars or below the plots.

In order to experimentally verify whether the fitness defects occurring under the SW condition in midsummer are attributable to high temperature, we allocated fresh egg masses of *N. viridula* to different constant temperature conditions at 25.0°C, 27.5°C, 30.0°C, 32.5°C, and 35.0°C using a long-day regime (16 h light/8 h dark) in laboratory climate incubators, reared them to adulthood, and measured their fitness parameters. As the rearing temperature increased from 25.0°C to 32.5°C, the survival rate declined, the nymphal period tended to be prolonged, and the adult body size became smaller. No nymphs reached the adult stage at the highest temperature of 35.0°C (Fig. 3). Whereas the insects reared at 25.0°C and 27.5°C exhibited a normal green color, the insects reared at 30.0°C and 32.5°C developed abnormal yellowish coloration in a temperature-dependent manner (Fig. 3E; see also Fig. S3 in the supplemental material).

The fitness defects were not observed under the QN conditions in midsummer (mean temperatures of 25.9 ± 1.6°C in July and 26.6 ± 1.8°C in August), whereas the fitness defects were prominent under the SW conditions in midsummer (mean temperatures of 28.6 ± 1.6°C in July and 29.4 ± 1.8°C in August) (Fig. 1D). These patterns are in agreement with the results obtained under the constant temperature conditions in the laboratory, wherein rearing at 25.0°C and 27.5°C caused no fitness defects, whereas rearing at 30.0°C resulted in remarkable defective phenotypes (Fig. 3). These results indicate that the fitness defects observed in *N. viridula* under the SW condition in midsummer were due to high-temperature stress.

Conventionally, although the underlying mechanisms were unknown, researchers have documented that symbiotic microorganisms of many insects are vulnerable to high temperature and that heat-challenged insects with a disrupted symbiotic system often exhibit retarded growth, elevated mortality, reduced fertility, abnormal body color, and other defective phenotypes (20, 23–27). In other animal-microbe symbiotic systems such as coral-dinoflagellate associations, it was also reported that high temperature conditions often lead to collapse of the associations (28–30). Recent advances in microbial genomics and ecology suggest that some, if not all, aspects of vulnerability of symbionts to high temperature are relevant to the general trend of degenerative evolution of their genomes (31, 32). In the aphid-*Buchnera* mutualism, a point mutation in the *Buchnera* genome was shown to be related to thermal tolerance of the host insect (33). As a consequence of increasing awareness of the potential impact of current and future climate change on ecological interactions among diverse organisms (1–4, 19), the possibility that heat-susceptible symbionts can be the “Achilles’ heel” of symbiont-dependent organisms under conditions of global warming has been pointed out (5, 6, 20). In this context, it is of interest to see how the QN, SW, and different constant temperature conditions influence the obligate gut symbiont of *N. viridula*.

### Suppressed symbiont population under simulated warming and high temperature conditions.

When the insects reared under the QN condition and the SW condition were subjected to symbiont quantification, remarkable differences between the treatments in symbiont titers were detected: whereas symbiont titers under the QN condition were constantly around 10^8^ to 10^9^ per insect from June to September, symbiont titers under the SW condition were drastically suppressed to 10^3^ to 10^4^ per insect specifically in midsummer (Fig. 2D and H). When the insects reared under different constant temperatures were subjected to symbiont quantification, notable differences in symbiont titers between the temperature conditions were also detected: whereas symbiont titers under the lower temperatures (25.0°C and 27.5°C) were around 10^8^ per insect, symbiont titers under the higher temperatures were significantly suppressed to 10^4^ to 10^6^ per insect at 30.0°C and to less than 10^4^ per insect at 32.5°C (Fig. 3D and H). These results indicate that the high temperature conditions (i.e., the SW condition in midsummer and the constant laboratory temperatures of 30.0°C and 32.5°C) resulted in not only the host’s fitness defects but also the symbiont’s population decline.

### Experimental suppression of symbiont population mimics defective phenotypes associated with simulated warming and high temperature conditions.

**FIG 4:**
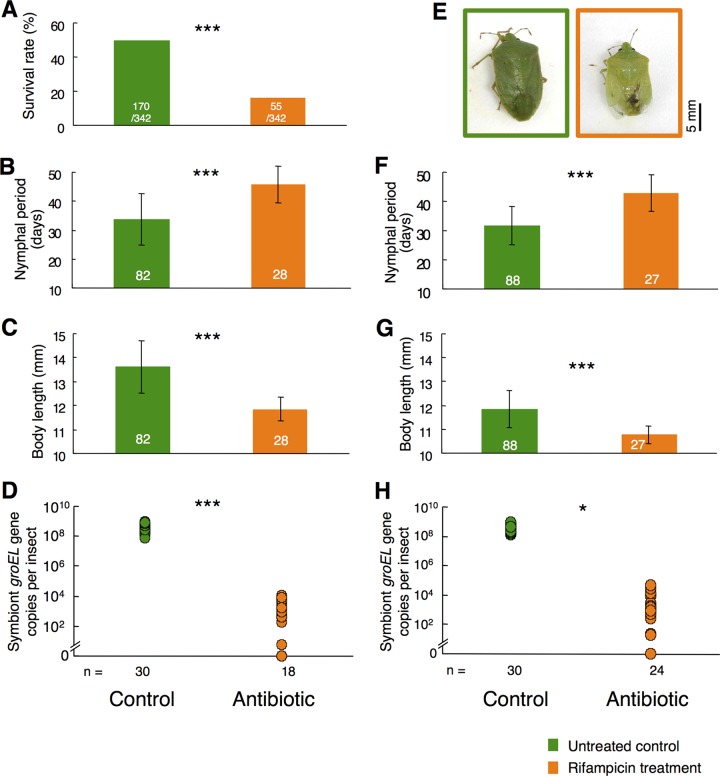
Effects of the low-dose antibiotic treatment on *N. viridula* and its symbiont population. (A) Survival rate (i.e., proportion of the eggs that reached adulthood, including both sexes). (B to E) Female insects: (B) nymphal period; (E) adult body length; (D) symbiont titer; (E) typical body color. (F to H) Male insects: (F) nymphal period; (G) adult body length; (H) symbiont titer. Green and orange indicate the nontreated control group and the rifampicin-treated group, respectively. Means and standard deviations are shown in panels B, C, F, and G. Asterisks indicate statistically significant differences (*, *P* < 0.01; ***, *P* < 0.0001). Sample sizes are shown on the bars or below the plots.

There are two alternative hypotheses to account for these patterns: (i) the high temperature conditions primarily affected the heat-vulnerable symbiont and the suppression of the obligate symbiont resulted in the host’s fitness defects or (ii) the high temperature conditions primarily damaged the host insect and, as a consequence of the disturbed physiology/performance of the host, the symbiont population was suppressed. In an attempt to test these hypotheses, we experimentally suppressed the symbiont population without heat stress (rearing at 25.0°C) using a low dose of antibiotic (0.001 mg/ml rifampicin) administered via drinking water to the host insects (see Fig. S1B in the supplemental material). Interestingly, the symbiont suppression by the antibiotic treatment, in which symbiont titers of around 10^8^ in the control insects were reduced to 10^4^ or less in the antibiotic-treated insects (Fig. 4D and H), resulted in induction of defective phenotypes for the host that were quite similar to those observed under the high temperature conditions, namely, reduced survival rate, prolonged nymphal period, smaller body size, and abnormal yellowish body color (Fig. 4; see also Fig. S4). These results strongly favor the former hypothesis in that the suppression of symbiont population without the heat stress can reproduce the host’s defective phenotypes. Meanwhile, the possibility should be kept in mind that the antibiotic treatment may have directly damaged not only the symbiont but also the host to some extent.

### Conclusion and perspective.

From all these results taken together, we conclude that high temperature conditions can suppress the obligate bacterial symbiont of *N. viridula*, thereby damaging the host insect indirectly and causing the heat-associated defective host phenotypes. Our laboratory data obtained under the strictly controlled environmental conditions (see Fig. 3 and 4) and our simulated warming data obtained in the seminatural settings (see Fig. 2) in combination with the field monitoring data that have been accumulated for decades (14–19) provide an insightful and integrated perspective as to how elevated temperature associated with global warming can impact insects and other organisms indirectly via affecting symbiotic/mutualistic microbial associates. For example, the northern distribution limit of *N. viridula*, which is temperature dependent and has recently been shown to be shifting northward in the Northern Hemisphere (14–19), might be substantially influenced not only by the host genotype but also by the symbiont genotype. How symbiont-mediated vulnerability to high temperature is relevant to the southern distribution limit of *N. viridula* is also of great interest and deserves future studies. It is conceivable, although speculative, that host-symbiont interactions, including symbiont-mediated heat vulnerability, could differ among local populations of *N. viridula* (14–18), a cosmopolitan species widely distributed from the temperate to tropical regions in the world (11). On account of the notorious pest status of *N. viridula*, such symbiont-mediated constraints on its distribution range are not only of ecological interest but also of practical relevance to, for example, prediction of its potential distribution range as an introduced insect pest.

Considering the omnipresence of microbial symbioses in nature, such phenomena, rather than being restricted to insect-microbe symbiotic associations, must also be found in other symbiotic systems such as coral-dinoflagellate photosynthetic associations, wherein high temperature-induced symbiosis collapse is called “coral bleaching” (28–30). In evaluating the ecological impact of global climate change, we must not forget to take into account the unseen microbial entities that are ubiquitously associated with a myriad of organisms and often play substantial biological roles therein.

## MATERIALS AND METHODS

### Insects.

*N. viridula* stinkbugs were collected around Osaka (Japan, 34.7°N, 135.5°E) in May 2009 and transferred to Kyoto (Japan, 35.0°N, 135.8°E), close to the current northern distribution limit of the species in Japan (16, 19). Culture stock of the insect was reared at 25°C under a long-day regime (16 h light/8 h dark) and supplied with dry soybean seeds (*Glycine max*), raw peanuts (*Arachis hypogaea*), and distilled water containing 0.05% ascorbic acid (18, 22). For rearing experiments, egg masses were collected from the culture stock, divided, and randomly allocated to the experimental conditions.

### Rearing under quasinatural and simulated warming conditions.

Climate warming was experimentally simulated by using a specially modified LH-350NSZ incubator (NK Systems, Osaka, Japan; for details, see reference 22), whose inner temperature was constantly controlled at 2.5°C higher than the outside temperature (Fig. 1D). On 5 June, 5 July, 5 August, and 5 September 2009, fresh egg masses from the culture stock were allocated to either the quasinatural (QN, i.e., outdoor) condition or the simulated warming (SW) condition (Fig. 1C). Figure S1A in the supplemental material depicts the experimental design. The temperature ranges under the QN condition were 22.9 ± 2.1°C in June, 25.9 ± 1.6°C in July, 26.6 ± 1.8°C in August, and 22.9 ± 2.1°C in September. The temperature ranges in the SW condition were 25.7 ± 2.1°C in June, 28.6 ± 1.6°C in July, 29.4 ± 1.8°C in August, and 25.6 ± 2.2°C in September. Under these conditions, insects were reared until adult emergence (for approximately 40 to 50 days). Insects were preserved in acetone after adult emergence until DNA extraction (34).

### Rearing under constant temperature conditions.

Fresh egg masses were randomly allocated to one of five different constant temperature conditions (25.0°C, 27.5°C, 30.0°C, 32.5°C, or 35.0°C) under the long-day regime and reared until the nymphs either molted to the adult stage or died. Insects were preserved in acetone after adult emergence until DNA extraction (34).

### Antibiotic treatment.

Hatchlings from the stock culture were divided into two groups and randomly allocated to either the antibiotic-treated condition or the nontreated control condition. In the former group, distilled water containing 0.001 mg/ml rifampicin was continuously supplied during the entire nymphal period until the hatchlings reached adulthood or died (up to 56 days after hatching). In the latter group, water without the antibiotic was supplied. In both treatments, water was replaced every third day. Figure S1B in the supplemental material depicts the experimental design. The insects were reared using a long-day regime at 25°C. Insects were preserved in acetone after adult emergence until DNA extraction.

### Microscopic observation.

The whole midgut was dissected from an adult insect using a pair of fine forceps under a binocular microscope in a plastic petri dish filled with phosphate-buffered saline (PBS; 137 mM NaCl, 8.1 mM Na_2_HPO_4_, 2.7 mM KCl, 1.5 mM KH_2_PO_4_ [pH 7.5]) and photographed by the use of a digital camera (EC3; Leica, Wetzlar, Germany) connected to a dissection microscope (S8APO; Leica, Wetzlar, Germany). The crypt-bearing midgut region (Fig. 1B) was collected from the whole midgut and homogenized in PBS. The suspension of crypt contents was stained with 4 µM 4′,6-diamidino-2-phenylindole (DAPI) (Invitrogen, Carlsbad, CA) and observed under an epifluorescence microscope (DMI 4000 B; Leica, Wetzlar, Germany).

### Fitness measurement.

The following fitness parameters were recorded for the experimental insects: survival rate (the proportion of eggs that reached adulthood); nymphal period (the number of days from egg hatching to adult emergence); and adult body length (the distance from the front edge of clypeus to the caudal tip). For the nymphal period and adult body length data, males and females were analyzed separately because females are larger than males in *N. viridula*. For survival rate data, males and females were analyzed together because nymphal sexing of *N. viridula* is difficult. The body length of the adult insects was measured by use of a digital caliper. For recording body color, the adult insects were photographed by using the same digital camera and the same light and distance settings.

### DNA extraction and sequencing.

Insects preserved in acetone were dissected in PBS with fine forceps and microscissors under the dissection microscope, and the symbiotic midgut region was isolated. Total DNA was extracted from the dissected symbiotic organ using a QIAamp DNA Mini Kit (Qiagen, Hilden, Germany). Successful extraction of DNA was confirmed by the use of a NanoDrop 1000 spectrophotometer (Thermo Fisher Scientific, Waltham, MA, USA) based on the optical density at 260 nm. A 1.6-kb region of the bacterial *groEL* gene was amplified by PCR with primers Gro-F1 (5′-ATG GCA GCW AAA GAC GTA AAT TYG G-3′) and Gro-R1 (5′-TTA CAT CAT KCC GCC CAT GC-3′) using AmpliTaq gold DNA polymerase (Applied Biosystems, Foster City, CA) and a temperature profile of 95°C for 10 min followed by 30 cycles of 95°C for 30 s, 55°C for 1 min, and 72°C for 2 min (35). Cloning and sequencing of the amplified product were performed as described previously (36).

### Quantitative PCR.

Real-time quantitative PCR was performed using SYBR green and an Mx3000P QPCR system (Stratagene, La Jolla, CA) essentially as described previously (37). On the basis of the 1.6-kb sequence of the symbiont *groEL* gene, specific internal primers MMAOgroF (5′-AAG CAA ATG ACG CTG CAG GAG-3′) and MMAOgroR (5′-GCT CAA CAG CAG CCA CAA CG-3′) targeting a 140-bp region for quantitative PCR were designed. The PCR mixture (in total, 20 µl) contained 2.0 µl of 10× PCR buffer (Applied Biosystems, Foster City, CA), 2.0 µl of GeneAmp deoxynucleoside triphosphate (dNTP) mix (Applied Biosystems, Foster City, CA) (2 mM [each] dATP, dTTP, dGTP, and dCTP), 1.2 µl of 25 mM MgCl_2_ solution (Applied Biosystems, Foster City, CA), 0.2 µl of SYBR green I (Molecular Probes, Eugene, OR, USA), 0.4 µl of primer mixture solution (5 µM [each] forward and reverse primers), 0.1 µl of AmpliTaq gold DNA polymerase (Applied Biosystems, Foster City, CA), 9.1 µl of distilled water, 1.0 µl of dimethyl sulfoxide, and 4 µl of extracted DNA. The PCR temperature profile was 95°C for 10 min followed by 40 cycles of 95°C for 10 s, 60°C for 15 s, and 72°C for 15 s. For calculation of the absolute numbers of the *groEL* gene copies, 10-fold serial dilutions of the target PCR product, ranging from 10^1^ to 10^7^ gene copies per reaction, were also quantified, and a standard curve was generated for the gene.

### Statistical analysis.

In the simulated warming experiments and the antibiotic treatment experiments, Fisher’s exact probability test (used to compare survival rates between treatments), Mann-Whitney *U* test (used to analyze duration of the nymphal period and body length), and median test (used to analyze symbiont quantity) were employed. In the constant-temperature rearing experiments, the Tukey-type multiple-comparison test for proportions (used to compare survival rates between treatments), Tukey HSD (honestly significant difference) multiple-comparison test (used to analyze the duration of the nymphal period and the body length), and Kruskal-Wallis test followed by multiple comparisons of mean ranks test (used to analyze symbiont quantity) were adopted. Multiple comparisons were corrected by the Bonferroni method. All statistical analyses were performed using the program Statistica 7.0 (StatSoft).

### Accession number(s).

The *groEL* gene sequence of the gut bacterial symbiont of *N. viridula* determined in this study has been deposited in the DNA Data Bank of Japan database under accession number AB979531.

## SUPPLEMENTAL MATERIAL

Figure S1  Experimental design. (A) In the simulated warming experiment, each egg mass of *N. viridula* was divided into two parts and allocated to either the quasinatural condition or the simulated warming condition (+2.5°C). (B) In the antibiotic experiment, each egg mass was divided into two parts and allocated to either the nontreated control group or the rifampin-treated group. Download Figure S1, PDF file, 0.6 MB

Figure S2  Effect of simulated warming on body coloration of *N. viridula*. Female and male insects were reared during August under the quasinatural condition or the simulated warming condition. Note that the insects reared under the simulated warming condition show smaller body size and abnormal body color. Download Figure S2, PDF file, 0.8 MB

Figure S3  Effect of elevated temperature on body coloration of *N. viridula*. Female and male insects were reared at one of five different constant temperatures (25.0°C, 27.5°C, 30.0°C, 32.5°C, and 35.0°C). Note that the insects reared at 30.0°C and 32.5°C show smaller body size and abnormal body color. No adult emergence was observed at the highest temperature (35.0°C). Download Figure S3, PDF file, 0.7 MB

Figure S4  Effect of antibiotic treatment on body coloration of *N. viridula*. Female and male insects were reared with distilled water (control) or rifampin-containing distilled water (rifampin). Note that the insects treated with the antibiotic show smaller body size and abnormal body color. Download Figure S4, PDF file, 0.8 MB
