# Supplementary material for: Collapse of Insect Gut Symbiosis under Simulated Climate Change
Source: mBio. 2016 Oct 4;7(5):e01578-16. doi: 10.1128/mBio.01578-16 (PMC5050343; doi:10.1128/mBio.01578-16)
Supplement: Figure S1 — Experimental design. (A) In the simulated warming experiment, each egg mass of N. viridula was divided into two parts and allocated to either the quasinatural condition or the simulated warming condition (+2.5°C). (B) In the antibiotic experiment, each egg mass was divided into two parts and allocated to either the nontreated control group or the rifampin-treated group. Download [file mbo005163011sf1.pdf]

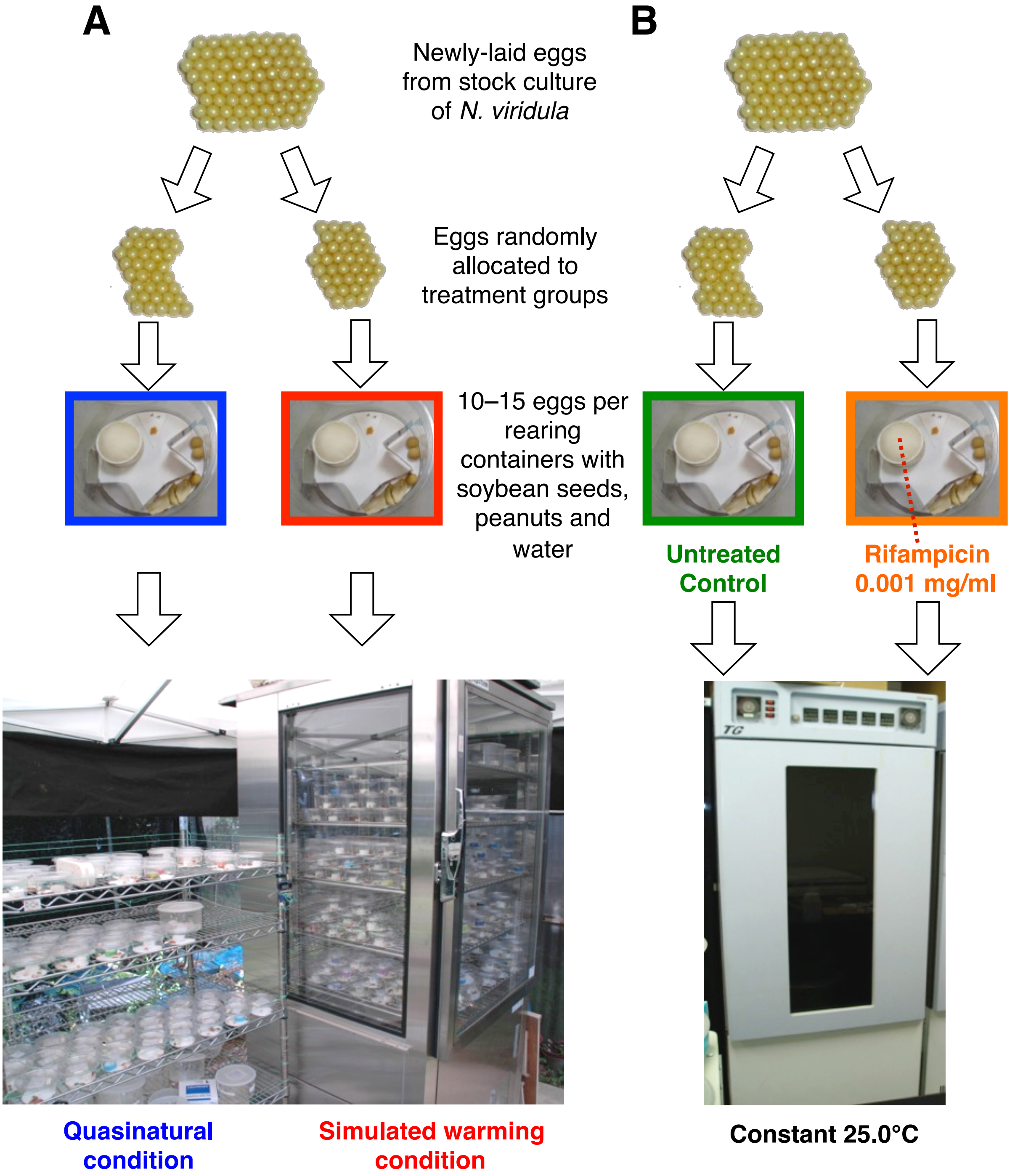


FIG S1 Experimental design. (A) In the simulated warming experiment, each egg mass of *N. viridula* was divided into two parts and allocated to either the quasinatural condition or the simulated warming condition (+ 2.5°C). (B) In the antibiotic experiment, each egg mass was divided into two parts and allocated to either the nontreated control group or the rifampicin-treated group.
