# Supplementary material for: Collapse of Insect Gut Symbiosis under Simulated Climate Change
Source: mBio. 2016 Oct 4;7(5):e01578-16. doi: 10.1128/mBio.01578-16 (PMC5050343; doi:10.1128/mBio.01578-16)
Supplement: Figure S2 — Effect of simulated warming on body coloration of N. viridula. Female and male insects were reared during August under the quasinatural condition or the simulated warming condition. Note that the insects reared under the simulated warming condition show smaller body size and abnormal body color. Download [file mbo005163011sf2.pdf]

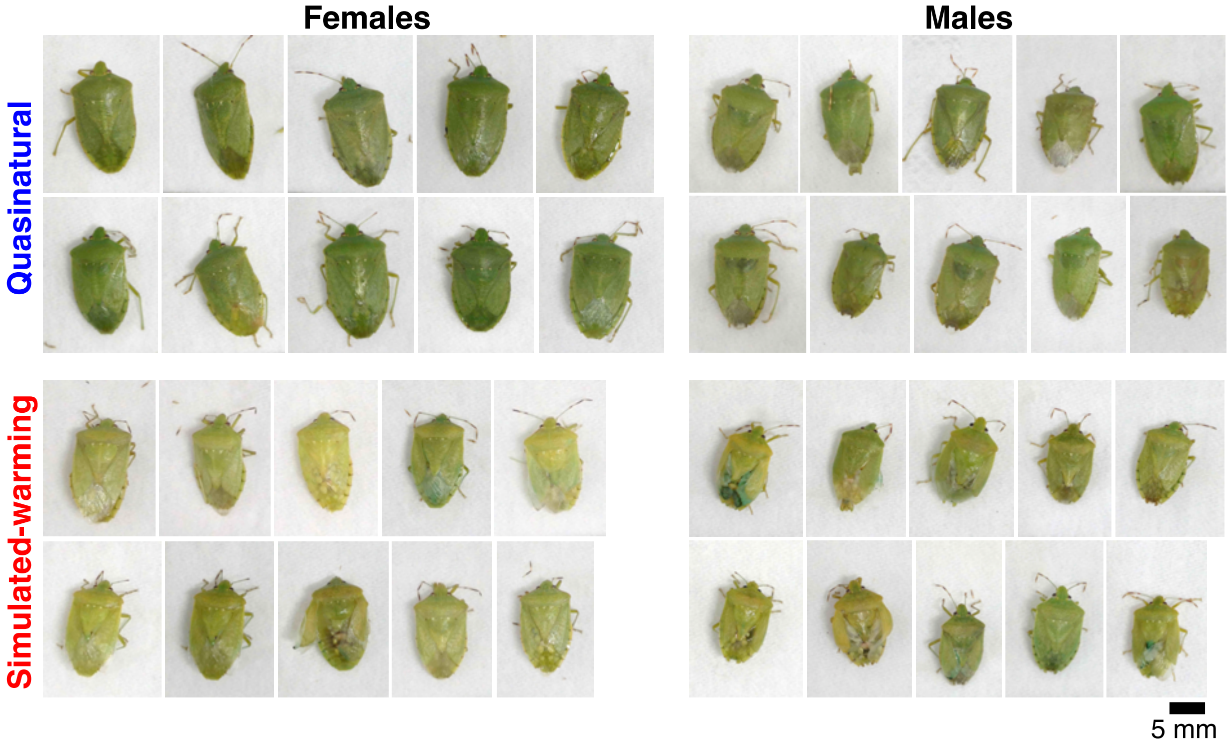

FIG S2 Effect of simulated warming on body coloration of *N. viridula*. Female and male insects were reared during August under the quasinatural condition or the simulated warming condition. Note that the insects reared under the simulated warming condition show smaller body size and abnormal body color.
