# Supplementary material for: Collapse of Insect Gut Symbiosis under Simulated Climate Change
Source: mBio. 2016 Oct 4;7(5):e01578-16. doi: 10.1128/mBio.01578-16 (PMC5050343; doi:10.1128/mBio.01578-16)
Supplement: Figure S3 — Effect of elevated temperature on body coloration of N. viridula. Female and male insects were reared at one of five different constant temperatures (25.0°C, 27.5°C, 30.0°C, 32.5°C, and 35.0°C). Note that the insects reared at 30.0°C and 32.5°C show smaller body size and abnormal body color. No adult emergence was observed at the highest temperature (35.0°C). Download [file mbo005163011sf3.pdf]

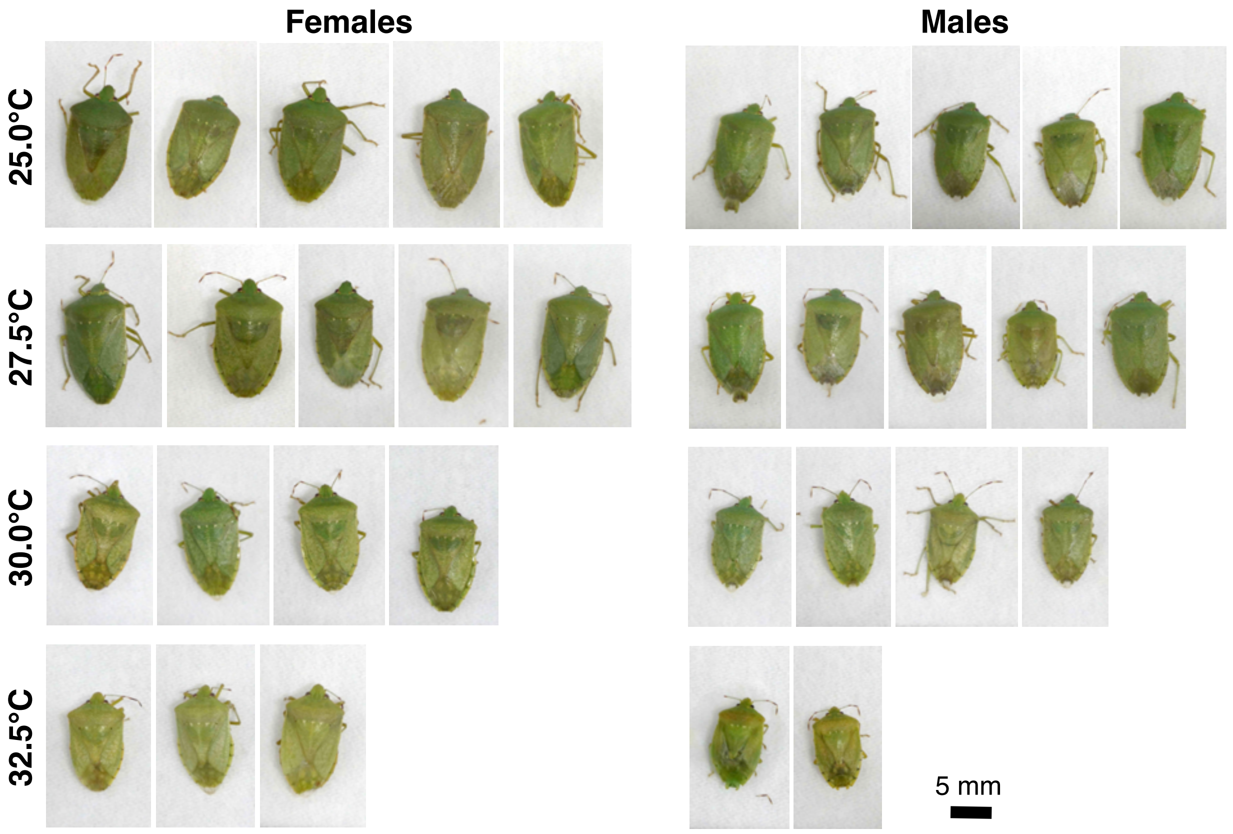

FIG S3 Effect of elevated temperature on body coloration of *N. viridula*. Female and male insects were reared at one of five different constant temperatures (25.0°C, 27.5°C, 30.0°C, 32.5°C, and 35.0°C). Note that the insects reared at 30.0°C and 32.5°C show smaller body size and abnormal body color. No adult emergence was observed at the highest temperature (35.0°C).
