# Supplementary material for: Collapse of Insect Gut Symbiosis under Simulated Climate Change
Source: mBio. 2016 Oct 4;7(5):e01578-16. doi: 10.1128/mBio.01578-16 (PMC5050343; doi:10.1128/mBio.01578-16)
Supplement: Figure S4 — Effect of antibiotic treatment on body coloration of N. viridula. Female and male insects were reared with distilled water (control) or rifampin-containing distilled water (rifampin). Note that the insects treated with the antibiotic show smaller body size and abnormal body color. Download [file mbo005163011sf4.pdf]

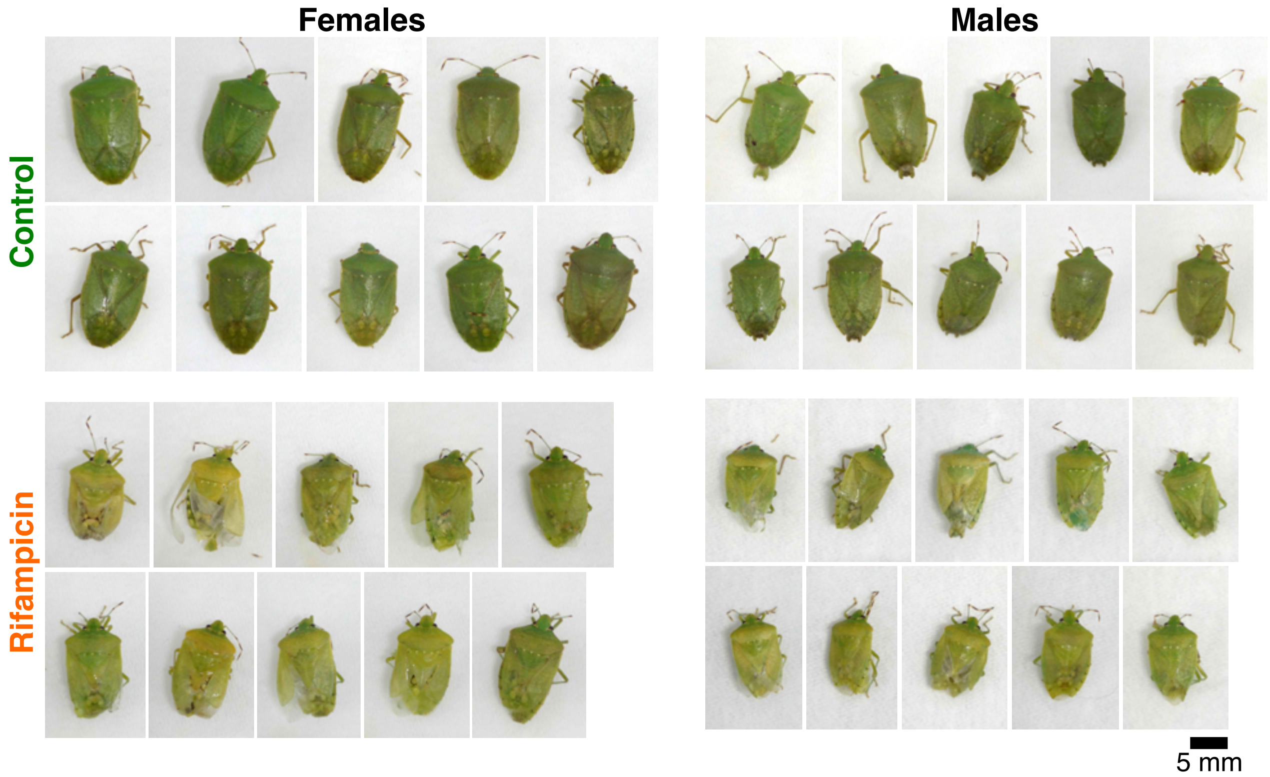

FIG S4 Effect of antibiotic treatment on body coloration of *N. viridula*. Female and male insects were reared with distilled water (control) or rifampicin-containing distilled water (rifampicin). Note that the insects treated with the antibiotic show smaller body size and abnormal body color.
